# A Case of Unilateral Renal Agenesis Detected by Abdominal Point-Of-Care Ultrasound

**DOI:** 10.7759/cureus.51453

**Published:** 2024-01-01

**Authors:** Takaaki Mori, Osamu Nomura, Naoaki Mikami, Hiroshi Hataya

**Affiliations:** 1 Department of Pediatric Emergency Medicine and Critical Care, Tokyo Metropolitan Children's Medical Center, Tokyo, JPN; 2 Department of Health Sciences Education, Hirosaki University, Hirosaki, JPN; 3 Department of Nephrology, Tokyo Metropolitan Children's Medical Center, Tokyo, JPN

**Keywords:** intussusception, urogenital anomalies, emergency department, children, ultrasound

## Abstract

As the interest in point-of-care ultrasound (POCUS) for investigating pediatric abdominal emergencies has been growing, an increasing number of literatures about abdominal POCUS has been published. We describe a noteworthy instance of a systematic approach using abdominal POCUS for detecting unilateral renal agenesis (URA) in previously healthy children with suspected intussusception. A previously healthy three-year-old girl was brought to our emergency department (ED) due to abdominal pain and bloody diarrhea. POCUS was performed to investigate the presence of intussusception. POCUS was able to rule out intussusception and detect URA. The investigation led the patient to a proper nephrology follow-up. When performing abdominal POCUS to evaluate gastrointestinal pathologies, it is important to pay attention to concomitant congenital anomalies of the kidney and urinary tract (CAKUT).

## Introduction

Congenital anomalies of the kidney and urinary tract (CAKUT) cause severe renal dysfunction, leading finally to end-stage renal disease (ESRD). Solitary functioning kidney is an important portion of CAKUT [1]. Unilateral renal agenesis (URA) is one of the major manifestations of this pathology and a major cause of solitary functioning kidneys, with an estimated worldwide incidence of one in 2000 births [2]. A significant number of CAKUT cases are typically diagnosed by antenatal, but several cases can be missed until later on in life when they present with hypertension, proteinuria, urinary tract infection, or renal dysfunction. Although some experts recommend routine check-ups for detecting renal anomalies in infants [3], there is no consensus guideline. By contrast, in recent years, POCUS has been introduced in pediatric emergency medicine, and its effectiveness has been proven for diagnosing various diseases [4]. In 2016, the guidelines of systematic approaches and standard documentation for pediatric POCUS were published [5]. In the present case, abdominal POCUS can detect URA in a previously healthy child who complained of bloody diarrhea.

## Case presentation

An otherwise healthy, three-year-old girl presented with abdominal pain and diarrhea four times a day eight hours before admission. The patient was brought to our ED because the last stool was bloody. On arrival, her vital signs were appropriate for her age, and her Glasgow Coma Scale score was 15. The patient denied nausea and vomiting. Her abdomen was soft and flat without tenderness or mass pulsation. No abnormal findings, including rectal bleeding in the genital region, were detected. POCUS was performed to investigate the presence of intussusception.

**Figure 1:**
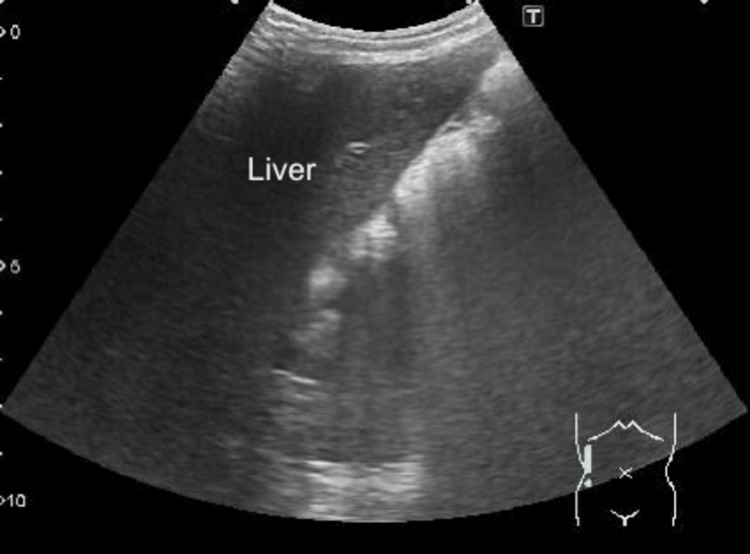
Abdominal POCUS finding (right abdominal short axis view) Ultrasonography demonstrated neither target nor pseudo-kidney signs in the ascending, transverse, and descending colons. Furthermore, no right kidney or free peritoneal fluid adjacent to the liver was detected in the right peritoneal cavity. POCUS: Point-of-care ultrasound.

**Figure 2:**
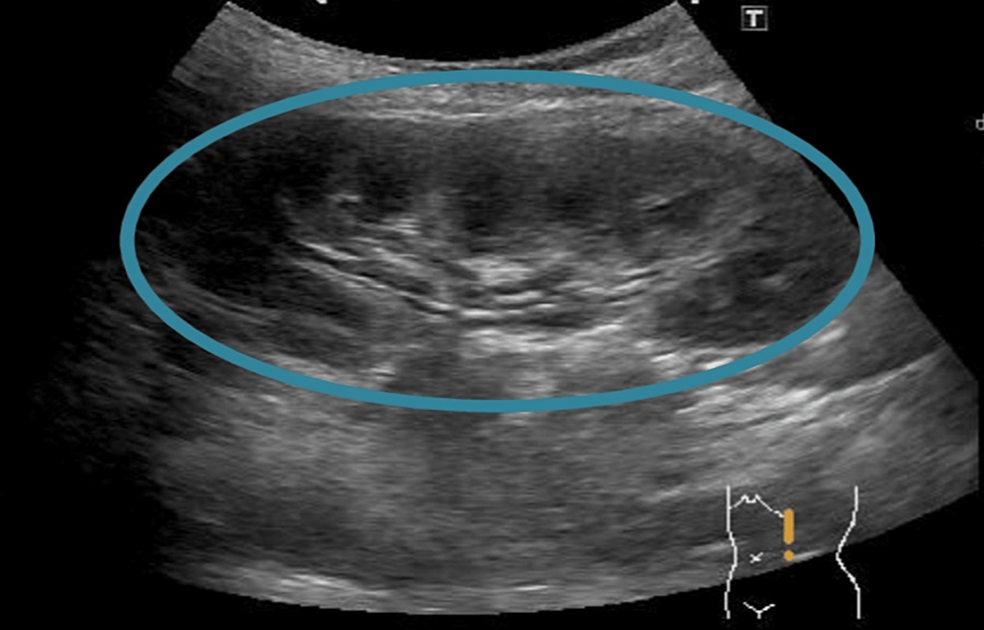
Abdominal POCUS finding (left abdominal short axis view) Ultrasonography showed a kidney with mild hydronephrosis (circle), but free peritoneal fluid was not detected. POCUS: Point-of-care ultrasound.

POCUS was performed using M-turboTM manufactured by FUJIFILM, SonoSite Inc., Japan, with a convex (2-5 MHz) and high-frequency linear transducer (6-13 MHz). The transducer was initially placed on both the upper quadrants and suprapubic legion to investigate the presence of intraperitoneal fluid, then on the right lower quadrant and the ascending colon, followed by the transverse colon and the descending colon to scan for intussusception. In the right peritoneal cavity, a kidney originally located caudally to the liver was not detected. In addition, there was neither a target sign nor a pseudo-kidney sign suggesting intussusception (Figure 1). On the left side, an enlarged kidney (9.0 cm in renal length) with mild hydronephrosis was found (Figure 2). In addition, intussusception-specific signs were also not recognized in the transverse to the descending colon.

Thus, although intussusception was excluded, URA was suspected, and the consultation with a nephrologist and further examination of her kidney and urinary tract were planned. The bloody stool disappeared after a day and was thought to have been caused by enterocolitis. On her initial nephrology follow-up, hypertension as well as proteinuria were not recognized. Ultrasonography performed by a radiologist confirmed URA and left hydronephrosis (Society of Fetal Ultrasound grade 1). An ectopic kidney was denied by 99m Tc-DMSA scintigraphy. Thereafter, although regular clinical monitoring for renal dysfunction and blood pressure was performed, no abnormal findings were detected during the one year following presentation.

## Discussion

This is a rare case report demonstrating that a systemic abdominal POCUS approach can detect CAKUT. POCUS has been gaining popularity in pediatric EDs in recent years [6,7]. As a result of the increasing number of reports demonstrating the effectiveness of POCUS for abdominal pathologies [8,9], guidance for the systematic scanning and documentation of POCUS has been published [5]. In accordance with the guideline, the scanning approach for intussusception in our hospital is to investigate the presence of intraperitoneal fluid and the whole colon from the ileocecum to the end of the descending colon. This approach can visualize the intestine as well as the kidney and urinary tract as the colon is adjacent to the kidney and urinary tract. Although the primary goal of POCUS in this case was to investigate the presence of intussusception, it functioned as a screening tool for CAKUT. Ultrasonography is a sensitive method for evaluating CAKUT, and not a few children with CAKUT are asymptomatic at the time of diagnosis [1]. Therefore, the systematic approach of abdominal POCUS is important not only for evaluating abdominal pathologies but also for detecting concomitant CAKUT.

CAKUT possibly causes renal dysfunction, and the solitary functioning kidney is an important facet of CAKUT [1]. Detecting the condition in childhood is challenging as the patients often exhibit no renal symptoms such as hypertension and proteinuria unless they occur concomitantly with other kidney and urinary tract anomalies [10]. Westland et al. demonstrated that the solitary functioning kidney with ipsilateral CAKUT showed a higher proportion of renal damage compared to the kidneys of children without ipsilateral CAKUT. However, renal damage still occurred in nearly one-third of the patients without CAKUT at 10 years of age [11]. In addition, a large study in the Netherlands showed that the mean age of the patients when their glomerular filtration rate began decreasing and hypertension began appearing was roughly nine years [12]. Thus, early detection and regular follow-up are paramount to managing renal dysfunction in its early stages.

URA is confirmed by diagnostic imaging such as ultrasonography and renal scintigraphy [1]. Although the procedures are generally used for patients with gastrointestinal symptoms, urinary tract infections, and abnormal urinalysis findings, there is no consensus on the indications. As ultrasonography is a safe and relatively less invasive diagnostic method, some experts have recommended using it in the routine screening of CAKUT in infants [3]. However, it is not cost-effective, and there is insufficient evidence of the efficacy of this screening method. Thus, abdominal POCUS should systematically be performed at least for the patients with gastrointestinal symptoms, and training in using the systematic approach should be implemented in pediatric EDs.

## Conclusions

We presented a case of a URA in a child detected by POCUS in the pediatric ED setting. The diagnosis of CAKUT is sometimes difficult, although routine check-ups for CAKUT were recommended in the infant period. POCUS can have the potential to detect concomitant CAKUT, optimizing further patient care. When performing abdominal POCUS in pediatric patients for the evaluation of gastrointestinal pathologies, a search for concomitant CAKUT should be considered.
